# Patellar tendon ossification after retrograde intramedullary nailing for distal femoral shaft fracture: A case report and review of the literature

**DOI:** 10.1097/MD.0000000000008875

**Published:** 2017-11-27

**Authors:** Lei Tan, Tiejun Wang, Yan-Hui Li, Tianye Yang, Baohui Hao, Dong Zhu, Da-Hui Sun

**Affiliations:** a Department of Orthopedic Traumatology; b Department of Cardiology and Echocardiography; c Department of Plastic and Cosmetic Surgery, The First Hospital of Jilin University. Changchun, China.

**Keywords:** heterotopic ossification, patellar tendon, retrograde femoral nailing

## Abstract

**Rationale:**

Retrograde femoral nailing was one of the most important treatment means for distal femoral shaft fracture. However, studies regarding heterotopic ossification of the patellar tendon after retrograde intramedullary nailing for distal femoral shaft fracture are limited. We herein present a rare complication, namely heterotopic ossification of the patellar tendon, after retrograde intramedullary nailing for displaced femoral shaft fracture.

**Patient concerns:**

We present a case of 25-year-old male with displaced femoral shaft fracture who was treated by retrograde intramedullary nailing.

**Diagnoses:**

During the period of follow-up, the patient developed symptomatic heterotopic ossification of the patellar tendon with extensively hard ossification area.

**Interventions:**

Open surgery was recommended, but the patient has refused further treatment.

**Outcomes:**

The patient resulted in pain and restricted the range of motion of the affected knee.

**Lessons:**

This case stresses the importance of longer-term follow-up and further attention into the possibility of heterotopic ossification of the patellar tendon.

## 1 Introduction

Initially, retrograde femoral nailing was used and developed for supracondylar femur fractures^[1]^; surgical indication of the application of this technique has been extended further to femoral shaft fractures, as well as intra-articular fractures, fractures involving the ipsilateral patella, and tibia or femoral neck.^[2–4]^ Complications come with the increasing application of retrograde femoral nailing for femur fractures, including decreased range of motion (ROM) of the knee, knee pain, nonunion, malunion, and arthrofibrosis. However, to date, literatures describing patellar tendon ossification after retrograde intramedullary nailing for distal femoral shaft fracture are limited.

Generally, heterotopic ossification is most commonly associated with musculoskeletal trauma such as acetabular fractures, elbow fracture dislocations, central nervous system disorders or injuries, severe burns, prolonged ventilation, and elective surgery such as total hip arthroplasty,^[5]^ antegrade femoral intramedullary nailing.^[6]^ Studies have demonstrated the highest incidence of heterotopic ossification at the hip joints,^[7]^ followed by the knee,^[8]^ elbow,^[9]^ and shoulder.^[10]^ Nonetheless, the occurrence of patellar tendon ossification is very rare. We herein present a rare complication, namely heterotopic ossification of the patellar tendon, after retrograde intramedullary nailing for displaced femoral shaft fracture.

## 2 Case report

**Figure 1:**
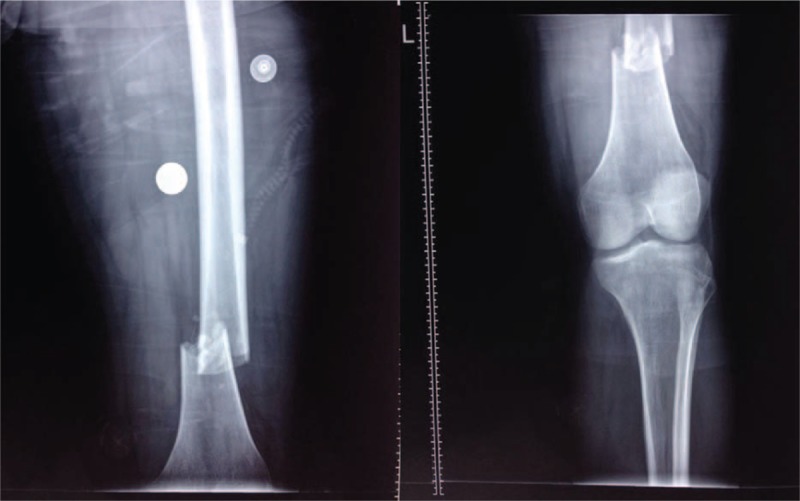
Anteroposterior (AP) view of the femur and knee: displaced distal femoral shaft fracture.

A 25-year-old man presented to our hospital which was involved in a high-energy traffic accident and suffered severe pain. Physical examination showed swelling and widespread tenderness over the left thigh. He had sustained a direct impact injury on his left thigh over the ground. No open wound and other evidence of associated injuries in the affected extremity were observed. There was no central or peripheral nerve injury. On the radiographs, a transverse fracture of the distal femoral shaft was found (Fig. 1) (AO type: 32-A3). Subsequently, an immediate internal fixation was performed with a reamed retrograde locked intramedullary nail after complete pre-anesthetic checkup; the femoral nail was inserted through a split tendinous approach. The patient tolerated the procedure well.

The patient was progressively rehabilitated without complications in our hospital for 7 days postoperatively and only bedside activities were instituted in his daily life for 1 month. During the early postoperative follow-up, no associated problems were observed. He was prescribed physical therapy to increase his ROM. Unfortunately, the patient moved to another province and was then lost to follow-up.

**Figure 2:**
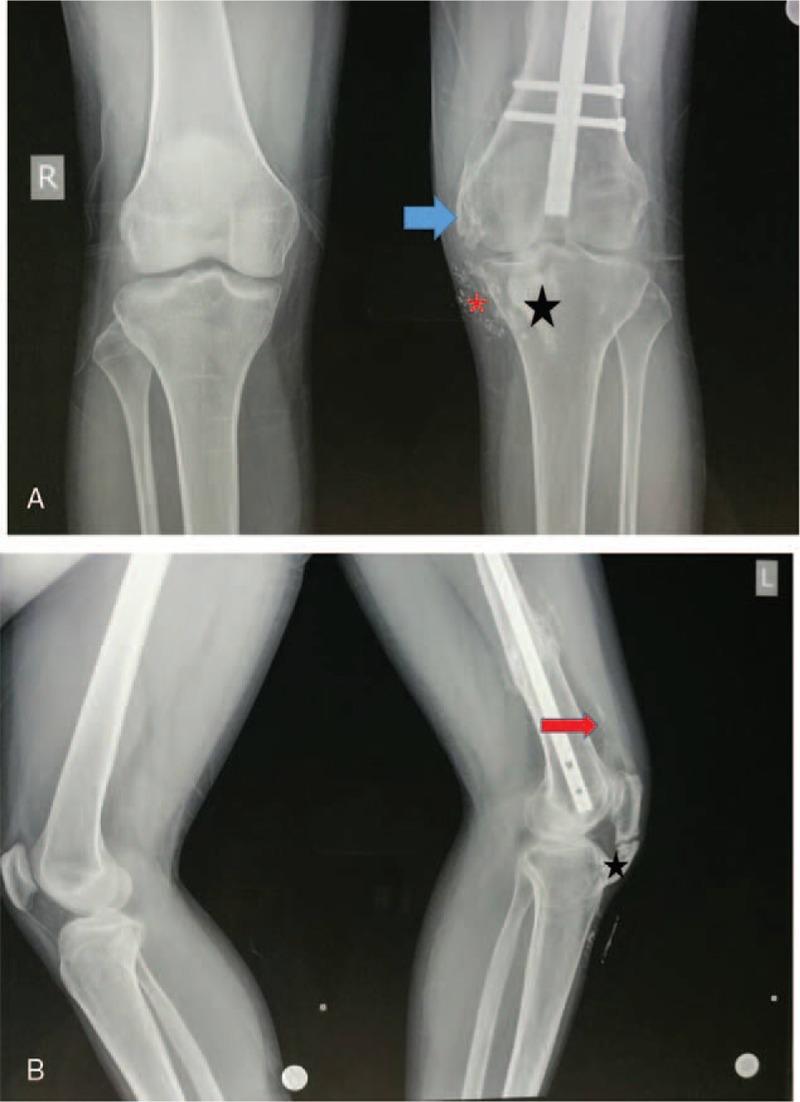
(A) Anteroposterior (AP) view of the knee: huge lesions of patellar ligament ossification (black star), medial collateral ligament (blue arrow), and small ossification scattered in the medial knee (red asterisk). (B) Lateral view of the knee: the fracture site was well healed with callus, huge lesions of patellar ligament ossification (black star), quadriceps tendon ossification (red arrow), and medial collateral ligament (blue arrow).

**Figure 3:**
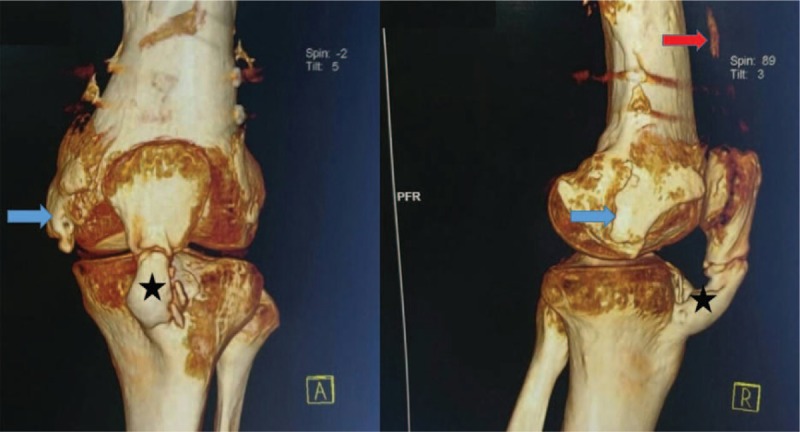
The computed tomography (CT) showed ossification of the patellar tendon (black star), quadriceps tendon ossification (red arrow), and medial collateral ligament (blue arrow).

Two years later, the patient returned to our department and reported persistent and progressive limited motion of the left knee. The patient did not experience other specified injuries of the affected knee after the surgery. He did not receive drug treatments or radiotherapy. Examination showed his previous wounds were healed. Two extensively hard ossification regions during palpation of the affected knee were observed. And one palpable tender mass was noticed within the area of the patellar tendon which was more evident in 50° knee flection. There was no evidence of erythema, swelling, and laxity. The ROM of the affected knee was restricted by pain at 0° to 50°. International Knee Documentation Committee (IKDC) score was 41.1. The radiological images revealed that the fracture ends were healed well with callus, which were accompanied with many ectopic ossifications including patellar ligament ossification, medial quadriceps tendon ossification, collateral ligament, and small ossification scattered in the medial of the knee (Fig. 2). The computed tomography (CT) revealed an area of ossification (6.8 × 3.1 × 1.2 cm) within the patellar tendon, which was well presented in the 3D reconstructions (Fig. 3). Blood tests including albumin, glucose, total proteins, lipids, creatinine, alanine amino transferase, blood urea nitrogen, aspartate amino transferase, sodium, alkaline phosphatase, potassium, bilirubin, calcium, and chloride were within normal ranges that exclude metabolic diseases. These findings revealed complete mature bone formation and then a diagnosis of “symptomatic heterotopic ossification within the patellar tendon” was rendered around knee joint. Therefore, open surgery was recommended, but the patient has refused further treatment. This study was approved by the institutional ethics board of the First Hospital of Jilin University and the written informed consent was obtained.

## 3 Discussion

The occurrence of patellar tendon ossification is very rare. Controversy exists in literature about the causes of the heterotopic ossification of patellar tendon but the affordable hypothesis is in favor of the traumatic etiology, which is usually associated with certain conditions such as total patellectomy during arthroplasty,^[11]^ anterior cruciate ligament reconstruction with patellar tendon autograft,^[12]^ conservative treatment for sleeve fractures of tibia tuberosity,^[13]^ knee injury without fracture,^[14]^ and intramedullary nailing of tibia fractures.^[15]^

Currently, retrograde locked intramedullary nails are known as the golden standard for displaced distal femoral shaft fractures and have a low complication rate that cannot be ignored.^[16]^ Court-Brown et al showed that knee stiffness is one of the most common complications, and reported 40.8% of their cohort having this complication.^[17]^ Although prominent/painful hardware often causes knee stiffness, heterotopic ossification is still a rare reported-cause of this problem. Only 2 cases of symptomatic heterotopic ossification of the patellar tendon were described by Horne et al in their literature^[18]^; these 2 patients developed knee pain and limitation of ROM which were consistent with radiographic evidence of patellar tendon ossification. All 2 cases had in common that they had closed traumatic brain injuries and were young (aged 20 and 45 years), while one of them needed mechanical ventilation for 3 weeks. And they were treated by patellar tendon splitting approaches. However, the difference of our study from them was that our case did not experience a head injury and mechanical ventilation.^[18]^ To our knowledge, this is the first reported clinical case of heterotopic ossification of patellar tendon occurring after retrograde intramedullary nailing for displaced femoral shaft fracture without contaminant injury.

Heterotopic ossification was caused by the independent differentiation of multipotent mesenchymal cells into osteoblastic cells induced by local and systemic factors.^[19]^ The formation of heterotopic ossification needs to satisfy 3 conditions, namely, osteogenic precursor cells, osteogenic inducers, and the osteogenic environment. The osteogenic precursor cells could have been seeded the tendon because of scattered bone debris and marrow reaming. Furlong et al^[20]^ reported that heterotopic ossification in the reamed group was significantly higher than those in the unreamed femoral nailing group. They found that heterotopic ossification was caused by the osteogenic reaming debris in these patients. Additionally, radical rehabilitation training may cause small micro tears in the tendon, resulting in bleeding and inflammation.^[21,22]^ We think that these hematomas and the inflammatory factor are the osteogenic inducer, which makes up the environment of the bone formation, results in forming further bone formation in the bone bed and tendon. However, the heterotopic ossification of the quadriceps tendon and the medial collateral ligament of the knee could not be explained by these theories.

Given all that, we suggest meticulous manipulation to avoid unnecessary future difficult problem. Treating measures are given below:

1. Minimally invasive technique with careful intraoperative hemostasis and postoperative drainage was recommended, which avoid bone debris and bone marrow into the soft tissue, rinse thoroughly, and prevent bone debris attached to the patellar ligament.
2. The patients were encouraged to take active exercise after the operation, so as to avoid brute forced passive exercise.
3. Once the heterotopic ossification occurs, indomethacin and radiotherapy can be used to prevent aggravation of ossification. The lesion can also be surgically removed to avoid knee joint mobility. In the case of a severe ossification of the ligament as in our case, surgical reconstruction may be performed immediately.

## 4 Conclusion

This case stresses the importance of longer-term follow-up; meticulous manipulation should be taken to avoid future difficult problem, and further attention into the possibility of heterotopic ossification of the patellar tendon after retrograde locked intramedullary nailing for distal femoral shaft fracture is required.
